# Expression of cluster of differentiation-95 and relevant signaling molecules in liver cancer

**DOI:** 10.3892/mmr.2014.3129

**Published:** 2014-12-22

**Authors:** XUMING WANG, KANGLAI WEI, QIONGGUANG ZHANG, SIEN ZENG, JING LIN, LI QIAO, LIJIANG LIU

**Affiliations:** 1 Department of Pathology, Guilin Medical University, Guilin 541001, P.R. China; 2 Department of Pathology and Pathophysiology, School of Medicine, Jianghan University, Wuhan Economy and Technology Development Zone, Wuhan, Hubei 430056, P.R. China; 3 Department of Pathology, School of Medicine, Jianghan University, Wuhan Economy and Technology Development Zone, Wuhan, Hubei 430056, P.R. China; 4 Department of Pathology, The First Affiliated Hospital of Guangxi Medical University, Nanning, Guangxi 530021, P.R. China; 5 State Key Laboratory of Virology, National Laboratory of Antiviral and Tumor of Traditional Chinese Medicine, Institute of Medical Virology, Research Center of Food and Drug Evaluation, School of Medicine, Wuhan University, Wuhan, Hubei 430056, P.R. China; 6 Office of Graduate Student Affairs, Guilin Medical University, Guilin 541001, P.R. China

**Keywords:** liver cancer, cluster of differentiation 95, caspase-8, caspase-3, poly(ADP-ribose) polymerase 1

## Abstract

The present study investigated the protein expression levels of cluster of differentiation (CD)95, caspase-8, caspase−3 and poly(ADP-ribose) polymerase 1 (PARP1) in liver cancer and its association with clinical pathological parameters. The results demonstrated that the expression of CD95 correlated with histological differentiation, liver cirrhosis, lymph node metastasis and distant metastasis (P<0.05), however, no correlations with gender, age, quantity of tumor nodules or T stage were observed (P>0.05). The expression of CD95 was upregulated using a plasmid, which led to an increase in the expression levels of caspase-8 and caspase-3 and a decrease in the expression of PARP1. Upregulation of CD95 also promoted the apoptosis of the liver cancer cells. These results indicated that CD95 was associated with liver cancer and promoted the apoptosis of liver cancer cells by caspase-8, caspase-3 and PARP1.

## Introduction

The cluster of differentiation (CD)95 antigen, also termed apoptosis antigen (APO)-1 or Fas, is a cell surface protein with a molecular weight of 200,000 Da, which differs to the molecular weight of the tumor necrosis factor (TNF) receptor (1). Anti-CD95 induces cell-death and its activity is indistinguishable from the cytolytic activity of TNF (1). A previous study observed that anti-CD95 induces apoptosis *in vivo*. Nanogram quantities of anti-CD95 completely inhibited the proliferation of cells containing APO-1 *in vitro*, characteristic of the process of programmed cell death or apoptosis (2). Complementary DNA (cDNA) encoding the Fas cell surface antigen were isolated from a cDNA library of human T-cell lymphoma KT-3 cells by Itoh *et al* (3) The nucleotide sequence of the cDNA revealed that the molecule coding for the Fas antigen determinant is a 319 amino acid polypeptide with a single transmembrane domain. The extracellular domain is rich in cysteine residues and is similar to that of human TNF receptors, the human nerve growth factor receptor and the CD40 human B cell antigen (3,4).

Apoptosis is the predominant form of eukaryotic cell death and occurs during tissue replacement, organ development, metamorphosis, tissue atrophy and tumor regression (4–6). Apoptosis is induced by a diverse range of agents, including glucocorticoids, cytostatic drugs, cytolytic cytokines, including TNF and lymph toxin, and in target cells of various killer cells, including cytotoxic T lymphocytes (4–6). The most prominent morphological features of apoptosis are chromatin condensation and membrane blebbing (4–6). In cells undergoing apoptosis, an endonuclease is induced, which cleaves the genomic DNA into polynucleosomal fragments and is observed on agarose gels as a ‘DNA ladder’ (4). Apoptosis and the Fas system are important in the process of converting liver cirrhosis into hepatocellular carcinoma. Downregulation in the expression of Fas and upregulation in the expression of Fas ligand in hepatocytes, and elevation of serum levels of Fas are important in tumor evasion from immune surveillance and in hepatic carcinogenesis (5,6). The present study investigated the relative expression of CD95 in liver cancer cells to determine whether there is a link between CD95 and liver cancer.

## Materials and methods

### Reagents

The rabbit polyclonal immunoglobulin G (IgG) anti-CD95 antibody (N-18; cat. no. sc-714), mouse monoclonal IgG_1_ anti-caspase-8 antibody (1.1.40; cat. no. sc-81656), rabbit polyclonal IgG anti-caspase-3 antibody (H-277; cat. no. sc-7148), goat polyclonal IgG anti-poly(ADP-ribose) polymerase 1 (PARP1) antibody (A-20; cat. no. sc-1562) and mouse monoclonal IgG_1_ anti-β-actin antibody (C4; cat. no. sc-47778) were purchased from Santa Cruz Biotechnology, Inc. (Santa Cruz, CA, USA). The radioimmunoprecipitation buffer and enhanced bicinchoninic acid assay kit were purchased from Beyotime Institute of Biotechnology (Jiangsu, China). Polyvinylidene difluoride (PVDF) membranes were purchased from EMD Millipore (Billerica, MA, USA). The LipoFiter™ Liposomal Transfection reagent was purchased from HanBio (Shanghai, China). The SP test kit and diaminobenzidine (DAB) colorization test kit were purchased from Beijing Zhongshan Golden Bridge Biotechnology, Co., Ltd. (Beijing, China). The AnnexinV-fluorescein isothiocyanate (FITC)/propidium iodide (PI) apoptosis detection kit was purchased from KeyGen Biotech, Co., Ltd. (Nanjing, China).

### Human liver cancer tissues

Frozen tumor samples and the corresponding normal liver tissues were obtained from 40 patients with gastric cancer, between 2006 and 2012 at Guangxi Medical University (Nanning, Guangxi, China) and paraffinembedded samples were obtained from 66 patients with gastric cancer, between 2006 and 2012 at Guangxi Medical University, Guilin Medical University, Guilin, Guangxi, China and Jianghan University, Wuhan, Hubei, China. Written informed consent was obtained from all patients. These samples were used following approval of the Institutional Review Board. The study was approved by the ethics committee of the School of Medicine, Jianghan University (Wuhan, China).

### Cell lines

The human HepG2 liver cancer cell line and the SGC7901 cell line were obtained from the Cell Center of Basic Medicine, Chinese Academy of Medical Sciences (Beijing, China).

### CD95 expression plasmid construction

The CD95 expression plasmid was donated by Dr JunFeng Zhang (Institute of Genetics and Developmental Biology, Chinese Academy of Sciences, Beijing, China). The total RNA was extracted from the SGC7901 cell line using TRIzol reagent (Invitrogen Life Technologies, Carlsbad, CA, USA) and the first strand cDNA was synthesized by reverse transcription using a ReverAid First Strand cDNA Synthesis kit (Thermo Fisher Scientific, Waltham, MA, USA) at 42°C, according to the manufacturer’s instructions. The entire CD95 coding region was amplified using the following primers: CD95, forward 5′-cactcgagCTTTCACTTCGGAGGATTGC-3′ (the added *Xho*I site is lowercase) and reverse 5′-gtgaattctGACCAAGCTTTGGATTTCATTTC-3′ (the added *Eco*RI site is underlined). The primers were designed using Primer 5.0 (Primer Biosoft International, Palo Alto, CA, USA) and were synthesized by the Sangon Biotech Co., Ltd. (Shanghai, China). Polymerase chain reactions were performed using a Ready-To-Use PCR kit (Tag DNA polymerase; no. SK2082, Sangon Biotech Co., Ltd) as follows: 94°C for 4 min, 30 cycles of 94°C for 30 sec, 60°C for 30 sec and 72°C for 70 sec with a final extension at 72°C for 10 min. The resulting fragment was digested using *Xho*I and *Eco*RI (Sangon Biotech Co., Ltd) and then subcloned into the pEgreen fluorescent protein (GFP)-N1, resulting in the N-terminal fusion to GFP. The resulting construct, pCMV IE: CD95-enhanced GFP (CD95-vector), was sequenced to confirm the in-frame fusion of CD95 and GFP and was used for transient expression in the HepG2 cells.

### Cell culture

All the cells were maintained in RPMI-1640 medium containing 10 % fetal bovine serum (FBS; Invitrogen Life Technologies) at 37°C in a 5 % CO_2_ atmosphere.

### Cell transfection

All the transfections were performed using LipoFiter™ Liposomal Transfection reagent, according to the manufacturer’s instructions. The HepG2 cells were plated (6×10^6^ cells) in 100 mm dishes and incubated overnight prior to replacing with fresh RPMI-1640 medium supplemented with 10% FBS. The cells were transfected with 4.6 μg of either CD95-vector or vector-control using 4.8 μl LipoFiter™ Liposomal Transfection reagent. The media was replaced 6 h after transfection and the cells were incubated at 37°C for 48 h. pEGFP-N1 was used as the vector-control. These transfected cells were used for subsequent experiments.

### Immunohistochemistry

Immunohistochemical staining was performed on 5 μm-thick tumor sections using a ‘two-step’ method. The tissue slides were de-paraffinized with 100% xylene for 10 min and rehydrated gradually in an alcohol series. The endogenous peroxidase activity was inhibited by incubation in a 3% hydrogen peroxide/methanol buffer for 10 min. Antigen retrieval was performed by immersing the slides in 0.5 mol/l ethylenediamine tetraacetic acid buffer (pH 8.0) for 10 min, followed by boiling in a waterbath for 25 min. The slides were rinsed in phosphate-buffered saline (PBS) and subsequently incubated with polyclonal anti-CD95 antibody (1:200) overnight at 4°C in a humidified chamber. Following incubation, the slides were washed three times with PBS containing 0.05% Tween-20 for 2 min each time. The slides were then incubated with 100 μl horseradish peroxidase polymer-anti-Mouse/Rabbit immunoglobulin G covering the tissue section in a moist chamber for 15 min. The slides were then washed three times, as previously, and the tissue was incubated for 5 min with 100 μl DAB chromogen. Following development of the appropriate color, the slides were washed gently under tap water for ~1–2 min, prior to counterstaining with 100 μl Mayer’s hematoxylin (Sangon Biotech Co., Ltd) covering the tissue completely for ~20 sec. The slides were rinsed thoroughly with tap water for ~1–2 min and subsequently incubated in PBS for 20 sec until the color turned blue. The slides were then rinsed with distilled water, followed by tap water.

The levels of CD95 staining were scored as follows: 0, no staining or staining observed in <10% tumor cells; 1+, faint/barely perceptible staining detected in ≥10% tumor cells; 2+/3+, moderate/strong staining, respectively, observed in ≥10% tumor cells. A score of 0/1+ was considered negative and a score of 2+/3+ was considered positive. The immunostained slides were evaluated independently by two pathologists in a blinded-manner. In the majority of cases, the results of the evaluation the two pathologists were identical; discrepancies were resolved by re-examination and consensus.

### Western blot analysis

For western blot analysis, the cells were washed with cold PBS and lysed in a lysis buffer containing 50 mM Tris-HCl (pH 8.0), 150 mM NaCl, 0.25 mM EDTA (pH 8.0), 0.1% SDS, 1% Triton X-100 and 50 mM NaF, supplemented with MS-SAFE™ Protease and Phosphatase Inhibitor Cocktail (1:100; Sigma-Aldrich, St. Louis, MO, USA) and phosphatase inhibitors (Sigma-Aldrich). The protein concentrations were determined using an Enhanced Bicinchoninic Acid Protein Assay kit (Beyotime Institute of Biotechnology). The cell lysates were mixed with loading buffer (Beyotime Institute of Biotechnology), separated using 12% SDS-PAGE gels and transferred onto a PVDF membrane (EMD Millipore, Billerica, MA, USA). The membranes were subsequently probed with various primary antibodies, appropriate secondary antibodies [goat anti-mouse IgG-horseradish peroxidase (HRP; sc-2005), goat anti-rabbit IgG-HRP (sc-2004) and donkey anti-goat IgG-HRP (sc-2020)] and visualized using enhanced chemiluminescence detection reagents (DNR Bio-Imaging Systems, Ltd., Jerusalem, Israel). The density of the protein bands were assessed using Totallab analysis software, version 2.01 (Nonlinear USA, Inc., Durham, NC, USA).

### Apoptotic assay

The cells were stained using an Annexin V-FITC apoptosis detection kit (Nanjing KeyGen Biotech, Co., Ltd., Nanjing, China) according to the manufacturer’s instructions, to detect early apoptotic cells (Annexin V+PI- events) and necrotic or late apoptotic cells (Annexin V+PI+ events) by flow cytometry. Briefly, the HepG2 cells were transfected with either the vector-CD95 or vector-control for 48 h. The cells were then collected and resuspended in the culture medium at a density of 1×10^6^ cells/ml, stained with 5 μL Annexin V-FITC and 5 μL PI in 300 μL binding buffer containing 10 mM HEPES, (pH 7.4), 140 mM NaOH and 2.5 mM CaCl_2_ according to the manufacturer’s instructions for 15 min at room temperature in the dark. Quantification of the apoptotic cells was assessed using a FACScan flow cytometer (Beckman Coulter, Brea, CA, USA).

### Statistical analysis

Statistical analysis was performed using SPSS 12.0 software (SPSS, Inc., Chicago, IL, USA). The data are expressed as the mean ± standard deviation of three replicates and were compared using Student’s t-test and analysis of variance. P<0.05 was considered to indicate a statistically significant difference. All experiments were performed at least three times to ensure reproducibility of the results.

## Results

### Association between the expression of CD95 and clinicopathological features in liver cancer

**Figure 1:**
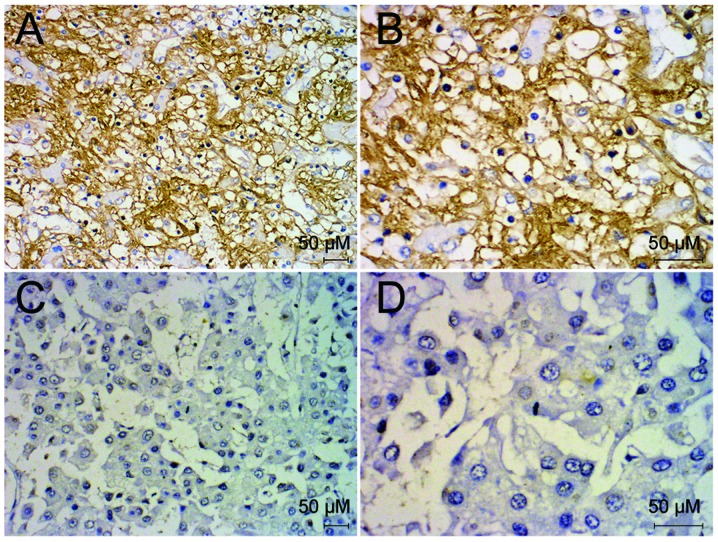
Immunohistochemical staining of liver cancer tissues. Representative images of the positive and negative CD95 staining. (A) Positive CD95 staining in the liver cancer cell membrane (magnification, ×100). (B) Positive CD95 staining in the liver cancer cell membrane (magnification, ×400). (C) Negative CD95 staining in the liver cancer cell membrane (magnification, ×100). (D) Negative CD95 staining in the liver cancer cell membrane (magnification, ×400).

The expression of CD95 was examined in the 66 liver cancer samples using immunohistochemistry. The expression of CD95 was detected predominantly in the cytoplasm of the liver cancer cells and at the plasma membrane, however, no expression was detected in the nuclei (Fig. 1). Positive expression of CD95 was detected in 17 of the liver cancer samples. The expression of CD95 correlated with histological differentiation, liver cirrhosis, lymph node metastasis and distant metastasis (P<0.05), however, no correlations with gender, age, quantity of tumor nodules or T stage were observed (P>0.05; Table I).

### Expression of CD95 is associated with the expression levels of caspase-8, caspase-3 and PARP1

**Figure 2:**
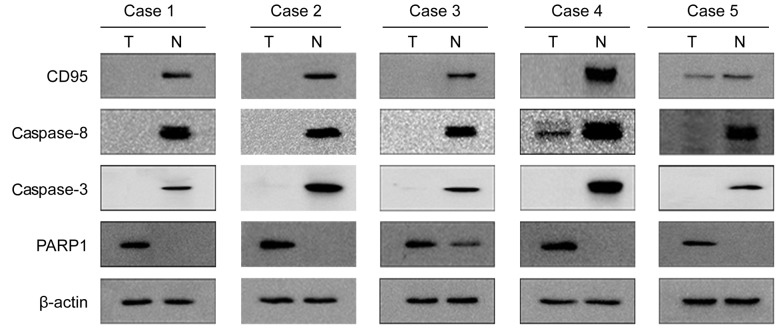
Western blot analysis of the expression levels of CD95, caspase-8, caspase-3 and PARP1 in specimens from liver cancer tissues and corresponding normal tissues. A representative western blot analysis result of five samples is shown. CD, cluster of differentiation; PARP, poly ADP ribose polymerase, T, liver cancer tissues; N, normal tissues.

The protein expression levels of CD95, caspase-8, caspase-3 and PARP1 in the tumor tissues and the corresponding normal tissues of 40 liver cancer samples were assessed by western blot analysis. The positive expression of CD95, caspase-8 and caspase-3 was detected in 14 (35%), 14 (35%) and 13 (32.5%) of the 40 tumor specimens, respectively. These levels were lower compared with those in the normal tissues, in which the positive expression of CD95, caspase-8 and caspase-3 was observed in 32 (80%), 33 (75%) and 36 (90%) specimens, respectively (Fig. 2). The expression of PARP1 was detected in 28 (70%) tumor specimens, compared with 16 (40 %) in the normal liver tissue specimens (Fig. 2). The expression of CD95 correlated with the expression of caspase-8, caspase-3 and PARP1 in the tumor specimens (Table II, P<0.01).

### Cell transfection

**Figure 3:**
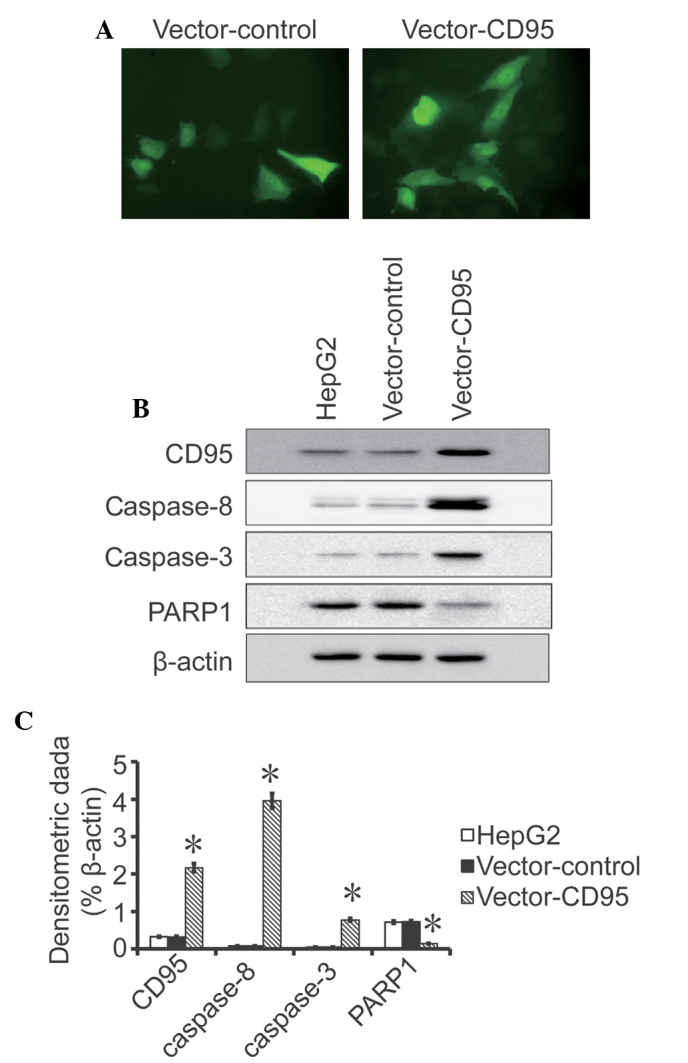
(A) Green fluorescence was observed at 24 h in the vector-control cells and the vector-CD95 cells (magnification, ×400). (B and C) Western blot analysis of the expression levels of CD95, caspase-8, caspase-3 and PARP1 in the recombinant liver cancer cell line. The data are expressed as the mean ± standard deviation from three independent experiments (^*^P<0.05, compared with HepG2). CD, cluster of differentiation; PARP, poly ADP ribose polymerase.

A positive GFP signal was detected in the High95 cells and in the control cells 24 h after transfection, which indicated that the transfection was successful (Fig. 3). Western blot analysis revealed that the expression of CD95 was higher in the High95 cells compared with the HepG2 cells and the control cells (P<0.05; Fig. 3). No difference was observed between the HepG2 cells and the control cells.

### Expression levels of CD95, caspase-8, caspase-3 and PARP1

**Figure 4:**
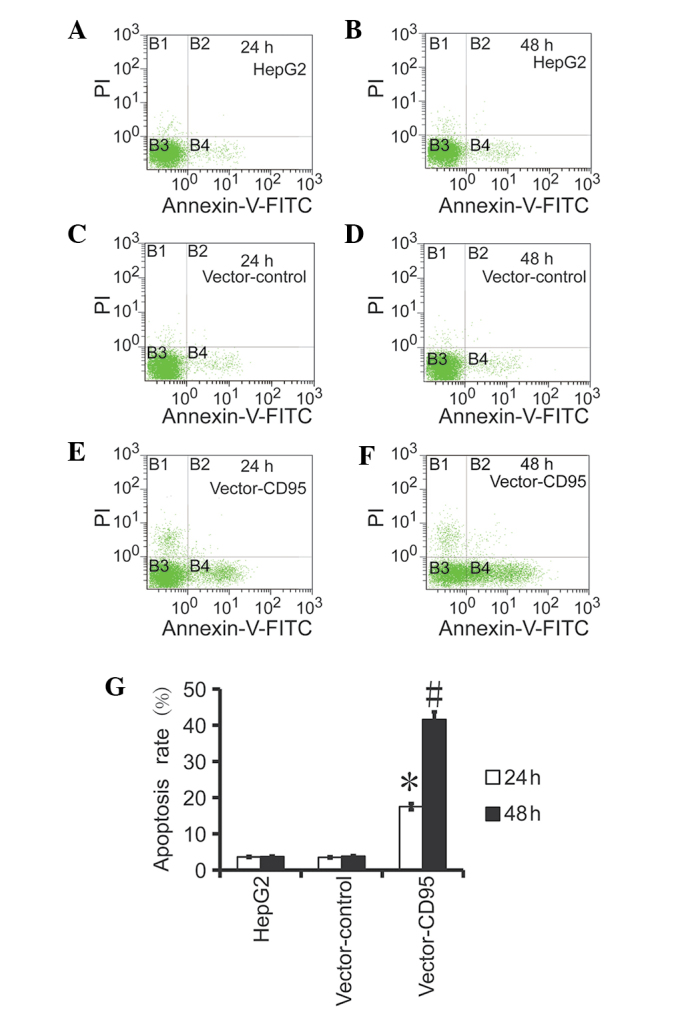
Apoptotic rate detected by flow cytometry. (A) Apoptotic rate at 24 h in the HepG2 cells. (B) Apoptotic rate of the HepG2 cells at 48 h. (C) Apoptotic rate of the thevector-control cells at 24 h and (D) 48 h. (E) Apoptotic rate of the vector-CD95 cells at 24 h and (F) 48 h. (G) Statistical analysis of the apoptotic rate in the HepG2 and recombinant liver cancer cell line. The data are expressed as the mean ± standard deviation from three independent experiments (^*^P<0.05 compared with the HepG2 cells). PI, propidum iodide; FITC, fluorescein isothiocyanate, CD, cluster of differentiation.

Western blot analysis indicated that the expression levels of CD95, caspase-8 and caspase-3 were higher in the High95 cells compared with the HepG2 cells and control cells (P<0.05; Fig. 4). The western blot analysis results also revealed that the expression of PARP1 was lower in the High95 cells compared with the HepG2 cells and control cells(P<0.05; Fig. 4). No difference was observed in the expression levels of CD95, caspase-8, caspase-3 and PARP between the HepG2 cells and control cells.

### CD95 promotes apoptosis in the HepG2 cells

The results of the flow cytometry revealed that the level of apoptosis in the High95 cells was higher compared with the HepG2 cells and the control cells (Fig. 5). Furthermore, the apoptotic rate was higher at 48 h compared with 24 h, which indicated that CD95 mediated apoptosis in the HepG2 cells.

## Discussion

CD95 is widely expressed in normal and diseased tissues and has been implicated in tumor progression in several types of cancer (5). Reduced expression levels of CD95 have been observed in a number of tumor types(7–12). The present study demonstrated that the expression of CD95 was lower in liver cancer tissues compared with normal liver tissues and correlated with histological differentiation, liver cirrhosis, lymph node metastasis and distant metastasis (P<0.05). Conversely, no correlations were observed with gender, age, quantity of tumor nodules or T stage, indicating that the expression of CD95 was associated with liver cancer.

In the present study, western blot analysis revealed that the expression of CD95 correlated with the expression levels of caspase-8, caspase-3 and PARP1. Caspase-8 is important in the CD95-mediated activation of the mitogen-activated protein kinases (MAPKs). A mechanism has been proposed, in which the catalytic activity and substrate specificity of caspase-8 are determined by the conformation and cleavage status of procaspase-8 (13). Procaspase-8 processing is required for the CD95-induced activation of MAPKs and conditions impairing MAPK activation are accompanied by reduced procaspase-8 processing (14). The activation of procaspase-8 is hypothesized to occur through an ‘induced proximity’ mechanism, involving the dimerization of procaspase-8 molecules, which facilitates activation through subsequent self-processing. This is in contrast to the executioner caspases, caspase-3 and caspase-7, which are constitutively dimeric and inactive due to the ‘strain’ caused by their short interdomain linker region on the active site and only become active on proteolytic cleavage (13). CD95 may rely exclusively on the activation of caspase-8 and the mitochondrial activation of caspase-3, which can process more procaspase-8 and, thus, propagate the amplification of the apoptotic signal (15). The present study revealed that the expression of caspase-8 was lower in the liver cancer tissues compared with the normal liver tissues.

Normal cells contain only a small quantity of caspases, in the form of inactive zymogens, and activated caspases are transformed to proteases via the catalytic activity of enzymes, which are capable of cleaving a number of substrate proteins resulting in apoptosis (16,17). Caspase-3 is activated by a series of cascade reactions until DNase is activated, which belongs to the Mg^2+^-dependent endonucleases and acts as an apoptotic factor (16). As caspase-3 is an effector caspase in apoptotic pathways, previous studies have hypothesized that a loss in the expression of caspase-3 may be important in the carcinogenesis of hepatocellular carcinoma (17). The present study revealed that the expression of caspase-3 was lower in the liver cancer tissues compared with the normal liver tissues.

PARP1 is a nuclear enzyme, which catalyzes PARP in target proteins in response to DNA damage and is considered to be important in DNA repair/recombination, cell death, cell proliferation and for stabilization of the genome (18). In several types of cancer, the expression of PARP1 is high and, therefore, inhibiting the expression of PARP1 may improve outcomes in patients with cancer (19–22). The present study demonstrated that the expression of PARP1 was higher in the liver cancer tissues compared with the normal liver tissues. In addition, the expression of PARP1 decreased as the expression of CD95 increased. The results demonstrated that inhibition of the expression of PARP1 may improve outcomes in patients with liver cancer.

## Figures and Tables

**Table I:** Association between the expression of CD95 and the clinicopathological features of liver cancer.

	Expression of CD95		
		
Factor	Positive (n)	Negative (n)	P-value
Gender			
Male	10	31	0.74
Female	7	18	
Age (years)			
≤50	5	16	0.80
>50	12	33	
Histological differentiation			
High	12	13	
Medial	2	8	0.002
Low	3	28	
Tumor nodules			
Single	6	20	0.68
Multiple	11	29	
Liver cirrhosis			
Positive	8	37	0.029
Negative	9	12	
T stage			
T1	5	7	
T2	6	11	0.064
T3	4	21	
T4	2	10	
N stage			
N0	13	19	0.016
N1–3	4	30	
M stage			
M0	16	32	0.047
M1	1	17	

CD, cluster of differentiation; n, number of cells.

**Table II:** Expression of CD95 is associated with the expression of caspase-8, caspase-3 and PARP1.

	Expression of CD95		
		
Factor	Positive (n)	Negative (n)	κ-value/P-value
Caspase-8			
Positive	12	2	κ=0.78
Negative	2	24	P<0.01
Caspase-3			
Positive	10	3	κ=0.609
Negative	4	23	P<0.01
PARP1			
Positive	3	25	P<0.01
Negative	11	1	

CD, cluster of differentiation; PARP1, poly(ADP-ribose) polymerase 1; n, number of cells.
